# Interdisziplinär ausgerichtetes Operationsspektrum in Kooperation von Viszeralchirurgie und Gynäkologie: Was sollte der (Allgemein‑/Viszeral‑)Chirurg wissen?

**DOI:** 10.1007/s00104-024-02033-w

**Published:** 2024-01-31

**Authors:** Gabriele Garlaschelli, Atanas Ignativ, Frank Meyer

**Affiliations:** 1 Klinik für Allgemein-, Viszeral-, Gefäß- und Transplantationschirurgie, Universitätsklinikum Magdeburg A. ö. R., Magdeburg, Deutschland; 2 Klinik für Gynäkologie und Geburtshilfe, Universitätsklinikum Magdeburg A. ö. R., Magdeburg, Deutschland

**Keywords:** Interdisziplinäre Zusammenarbeit, Interdisziplinäres Fallmanagement, Gynäkologische Krankheitsbilder, Intestinale Resektion, Chirurgische/operative Komplikationen, Interdisciplinary cooperation, Interdisciplinary case management, Gynecological diseases, Intestinal resection, Surgical/operative complications

## Abstract

**Ziel:**

Die vorliegende Kurzübersicht soll in prägnanter Form aus bevorzugt operativer Sicht die wichtigsten gynäkologischen Fragestellungen erläutern, welche auch für Allgemein- und ViszeralchirurgInnen relevant sein können, sowie wesentliche gynäkologische Aspekte primär viszeralchirurgischer Krankheitsbilder aufzeigen.

**Methode:**

Narrative Übersicht zum Thema der gynäkologisch/allgemein- und viszeralchirurgischen interdisziplinären Kooperation mithilfe von PubMed® sowie der Cochrane Llibrary unter Verwendung von Suchbegriffen wie „operative profile of abdominal surgery and gynecology“, „interdisciplinary surgery aspects of gynecology/abdominal surgery“ sowie „interdisciplinary surgical approach – surgical complication“.

**Ergebnisse (Eckpunkte):**

Viele primär gynäkologische Krankheitsbilder können, allein durch die engen anatomischen Verhältnisse, auch an abdominellen Organen auftreten. Ebenso können primär viszeralchirurgische Pathologien auch eine Involvierung der gynäkologischen Organe bedingen. Hierdurch kann eine intraoperative Zusammenarbeit notwendig werden. Auch prä- und postoperativ kann aufgrund diagnostischer Unsicherheit oder im Rahmen von Komplikationen eine Interdisziplinarität gefordert sein. Dabei können ein fächerübergreifendes Wissen der therapierenden ÄrztInnen sowie die enge Kooperation der gefragten Fachrichtungen das Outcome der betroffenen Patientinnen verbessern.

**Schlussfolgerung:**

Viele Krankheitsbilder erstrecken sich nicht nur bis an die Grenze der einzelnen Fachbereiche, sondern können darüber hinaus auch weitere Systeme betreffen. Für eine optimale Therapie ist es daher notwendig, auch solche Aspekte der Pathologien zu kennen sowie strukturierte Abläufe der interdisziplinären Kooperation zu etablieren.

## Abstract (en)

**Aim:**

This short overview aims to concisely outline the most important gynecological issues from a predominantly operative point of view, which could also be relevant for general and abdominal surgeons as well as important gynecological aspects of primarily visceral surgical pathologies.

**Method:**

Narrative review on the topic of interdisciplinary cooperation in gynecological/general and abdominal surgery through the use of PubMed® as well as the Cochrane Library with search terms, such as “operative profile of abdominal surgery and gynecology”, “interdisciplinary surgery aspects of gynecology/abdominal surgery” as well as “interdisciplinary surgical approach−surgical complication”.

**Results (main points):**

As the close anatomical relationship suggests, numerous primarily gynecological pathologies can also occur in abdominal organs. Likewise, predominantly surgical pathologies can result in involvement of gynecological organs. This can make an intraoperative collaboration necessary. In addition, as a result of diagnostic uncertainty or within the context of complications, interdisciplinary collaboration can also be required preoperatively and postoperatively. Multidisciplinary knowledge as well as close cooperation of the involved specialties can improve the outcome of affected patients.

**Conclusion:**

Many pathologies extend not only to the boundaries of an individual discipline but can also affect physiological systems exceeding those limits. Therefore, for an optimal treatment it is necessary to be aware of such aspects of the diseases and to establish structured procedures for interdisciplinary cooperation.

## Hintergrund

Die mehrheitlich stürmische Entwicklung der diversen medizinischen Fachdisziplinen des humanmedizinischen Spektrums hat zu einer Vielzahl an Überlappungen unterschiedlicher Fächer im klinischen Fallmanagement geführt, die eine fachübergreifende Verständigung geradezu herausfordern.

Die Entwicklungsgeschichte der Gynäkologie lässt sich über Jahrtausende zurückverfolgen. So lässt sich in etwa das *Kahun Papyrus* des antiken Ägyptens, welches sich mit Schwangerschaftsfeststellung, Kontrazeption sowie einer Vielzahl gynäkologischer Erkrankungen beschäftigt, auf 1900 vor Christus zurückdatieren. Um das 3. Jahrhundert vor Christus findet sich schließlich mit dem *Corpus Hippocraticum* „zweifelsohne das Fundament der westlichen Medizin“. Von dieser Kollektion aus Texten können laut einigen Berechnungen etwa ein Viertel dem Fachgebiet der Gynäkologie zugeordnet werden [53]. Auch in der jüngeren Geschichte blieb die Gynäkologie weiterhin ein Gebiet der aktiven Forschung. So wurden beispielsweise laparoskopische Untersuchungs- sowie Operationstechniken maßgeblich durch GynäkologInnen mitentwickelt, wohingegen diese von vielen chirurgischen KollegInnen zunächst für unseriös gehalten wurden [18, 63]. Trotz der jahrhundertelangen und zum Teil auch eigenständigen Entwicklung dieser Fachrichtung gibt es einige bedeutsame Überlappungen mit der Allgemein- und Viszeralchirurgie. So sind Patientinnen beispielsweise auf der Grundlage bildgebender pathologischer Befunde präoperativ nicht immer sicher einer Fachrichtung zuzuordnen [26, 33]. Zudem kann auch intraoperativ eine interdisziplinäre Kooperation indiziert sein [22, 96]. Weiterhin können intra- sowie postoperative Komplikationen das Hinzuziehen von KollegInnen anderer Fachrichtungen notwendig machen [94]. Hierdurch erklärt sich, dass die Allgemein- und Viszeralchirurgie oftmals zum bevorzugten Ansprechpartner für bauchchirurgische Fragestellungen bei primär zunächst gynäkologischen Patientinnen werden kann.

### Ziel.

Die vorliegende kompakte Kurzübersicht soll in prägnanter Form gynäkologisch-viszeralchirurgisch interdisziplinär relevante Fragestellungen aus bevorzugt operativer Sicht charakterisieren und hierdurch die Relevanz der interdisziplinären Kooperation erläutern.

## Methode

Narrative Übersicht, basierend auf selektiven Referenzen der medizinisch-wissenschaftlichen Literatur und einschlägigen klinischen Erfahrungen aus der täglichen gynäkologischen, überlappend allgemein‑/viszeralchirurgischen Praxis mit operativer Relevanz unter Nutzung der folgenden Suchbegriffe in PubMed® sowie der „Cochrane Library“: Operative profile of abdominal surgery and gynecology – Interdisciplinary surgery aspects of gynecology/abdominal surgery – Interdisciplinary surgical approach – Surgical complications unter bevorzugter Heranziehung und Berufung auf Literatur der letzten 10 Jahre sowie Bezug auf aktuelle Leitlinien. Insbesondere soll auf Quellen Wert gelegt werden, die interdisziplinäre Aspekte und gegebenenfalls die Relevanz der Interdisziplinarität hervorheben.

## Ergebnisse (Eckpunkte)

### Erkrankungsbilder mit gynäkologischem Fokus

#### Maligne gynäkologische Erkrankungen

##### Ovarialkarzinom.

Trotz erheblicher Fortschritte der letzten Jahre in der Behandlungsqualität selbst fortgeschrittener Ovarialkarzinome bleiben diese hinsichtlich ihres adäquaten diagnostischen und therapeutischen Managements weiterhin eine anspruchsvolle Erkrankung [35, 80]. Dabei steht die Operation (Op) mit dem Ziel der makroskopisch und mikroskopisch totalen Tumorresektion (R0) nach wie vor im Zentrum der therapeutischen Bemühungen mit kurativer Intention. Dies stellt den wichtigsten prognostischen Faktor beim Ovarialkarzinom auch im fortgeschrittenen Stadium dar. Im Falle einer diffusen Tumoraussaat und bei nicht bestehender Möglichkeit der vollständigen Resektion sollte die erreichbare Tumorrestmasse auf möglichst kleine Areale beschränkt werden, welche nicht mehr palpabel sind und die beste Voraussetzung für eine anschließende (additive/palliative) Chemotherapie bieten [12, 43, 80].

Die Rate an kompletten Tumorresektionen konnte in den letzten Jahren in Deutschland signifikant gesteigert werden [80]. Erschwerend ist jedoch, dass sich durch die anatomischen Verhältnisse im kleinen Becken insbesondere zwischen Rektum und inneren Genitalien Konglomerattumoren ausbilden können, die sich nicht selten organüberschreitend manifestieren. Um das Ziel der maximalen Tumorresektion zu erreichen, kann dann ein radikales Tumordebulking inklusive Darmresektion bis hin zu einer pelvinen Exenteration (siehe unten) nötig werden [39, 42]. Die am häufigsten durchgeführte Darmresektion im Rahmen ovarialkarzinombedingter Operationen ist dabei die des Rektosigmoideums, gefolgt von der Dünndarmresektion und der rechtsseitigen Hemikolektomie [42].

Neben einer Beteiligung des Darmes finden sich im Rahmen fortgeschrittener Ovarialkarzinome häufig auch Fernmetastasen, von denen der Großteil wiederum intraabdominell lokalisiert ist. Das am häufigsten betroffene solide Organ ist hierbei die Leber (Tab. 1; [35]). Neuere Studien konnten zeigen, dass selbst bei Ovarialkarzinomen mit hepatischer Metastasierung, welche typischerweise mit einem fortgeschrittenen Tumorgeschehen assoziiert sind, eine vollständige Resektion möglich sein kann [22].

**Table 1:** 

Prozentuale Verteilung der Lokalisation von Metastasen	Primarius	
	Ovar	Uterus
Lunge	38,4	61,8
Leber	56,8	21,9
Knochen	3,5	13,2
Gehirn	1,3	3,1

**Figure 1:**
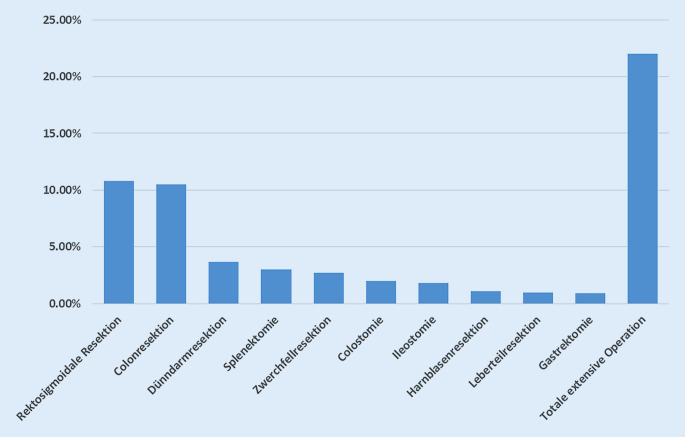

Insgesamt wird bei etwa 22 % der Patientinnen mit operativ therapiertem Ovarialkarzinom eine extensive Op mit Involvierung der abdominellen Organe nötig [96]. Abb. 1 zeigt dabei die Häufigkeit verschiedener viszeralchirurgischer Interventionen in der operativen Versorgung des Ovarialkarzinoms. Eine interdisziplinäre Beurteilung mit anschließender multiviszeraler Operation ist hierfür die Voraussetzung [22].

Zusätzlich zur reinen chirurgischen Resektion kann bei weit ausgedehnten Befunden, insbesondere nach präoperativ gutem Ansprechen auf eine neoadjuvante Chemotherapie, auch die Nutzung einer hyperthermen intraperitonealen Chemotherapie (HIPEC) erwogen werden. Das typischerweise im Rahmen viszeralchirurgischer Interventionen genutzte Verfahren ist beim Ovarialkarzinom noch kein Standard, konnte aber in Studien unter gut definierten Voraussetzungen ansprechende Ergebnisse zeigen und wird weiter erforscht [5, 31, 90, 99].

Unter den malignen gynäkologischen Erkrankungen ist daher speziell beim Ovarialkarzinom häufig ein interdisziplinäres Vorgehen im Rahmen der Behandlung notwendig [11, 17, 22], um die operative Expertise für ein bestmögliches operatives Outcome zu bündeln. Insbesondere um die versorgenden Gefäße zu erhalten und eine funktionelle Rekonstruktion durchzuführen, sollte hierzu möglichst frühzeitig chirurgische Unterstützung hinzugezogen werden [43]. So beschreibt auch die aktuelle S3-Leitlinie zur Therapie maligner Ovarialtumoren: „Ein interdisziplinäres Vorgehen mit Gynäkoonkologie und z. B. Viszeralchirurgie […] ist bei ausgedehnt multiviszeralen Operationen häufig notwendig“ [93].

##### Endometriumkarzinom.

Beim Endometriumkarzinom handelt es sich um die häufigste maligne Erkrankung des weiblichen Genitale und die fünfthäufigste Krebserkrankung bei Frauen [29]. Eine Beteiligung extragenitaler Organe kann durch die Ausbildung von Fernmetastasen, welche in häufigster Lokalisation in Lunge und Leber vorkommen (Tab. 1), durch Infiltration *per continuitatem*, etwa des Intestinums, aber auch durch die Entstehung enterovaginaler Fisteln bedingt sein [28, 35, 52]. Das operative Therapieziel zur Verbesserung des Überlebens auch in fortgeschrittenen Stadien ist die Erreichung eines R0-Resektionsstatus. Meist erfolgt hierzu eine totale Hysterektomie mit beidseitiger Adnexexstirpation, wobei auf letztere in einigen Fällen verzichtet werden kann [8, 27, 28, 52]. Entsprechend der Tumorausdehnung können dabei auch Resektionen extragenitaler Organe wie des Omentum majus oder von Darmteilen nötig werden [59]. In einigen Fällen kann sogar eine deutlich radikalere Op-Technik gewählt werden – so kann beispielsweise bei Frauen mit rezidivierendem Endometriumkarzinom auch eine pelvine Exenteration erwogen werden. Gleichwohl eine systematische Literaturanalyse der „Chochrane Collaboration“ keine den Einschlusskriterien entsprechende kontrollierte Studie zur Beurteilung der Durchführung dieser Intervention bei Patientinnen mit Rezidiven gynäkologischer Malignome eruieren konnte, stützen einige Studien eher niedrigeren Evidenzgrades den Nutzen der pelvinen Exenteration auch bei gynäkologischen Malignomen, sodass diese Therapieoption auch Einzug in die aktuellen Leitlinien fand [4, 23, 28, 69, 81].

#### Endometriose mit Beteiligung viszeraler Organe

Während Endometrioseherde am häufigsten an gynäkologischen Organen im kleinen Becken zu finden sind, können sie seltener auch diverse andere Lokalisationen wie beispielsweise an Darm, Pleura oder Perikard aufweisen [37, 45]. Einen besonderen Stellenwert hat die Endometriose in erster Linie durch ihre hohe Prävalenz, welche in der allgemeinen weiblichen Bevölkerung bei 6–10 % liegt [37]. Dabei kann die Erkrankung asymptomatisch verlaufen oder auch mit Dysmenorrhö, Dyspareunie und/oder Infertilität einhergehen. Abhängig von der Lokalisation der Endometrioseherde können auch weitere spezifische Symptome wie ein katamenialer Pneumothorax oder Dyschezie auftreten [82, 92]. In letzteren Fallkonstellationen kann sich auch die Differenzialdiagnose zur Colitis ulcerosa oder dem Reizdarmsyndrom schwierig gestalten [82].

Während die Erstlinientherapie der Endometriose meist medikamentös ist, kommt eine operative Therapie insbesondere bei Frauen mit schweren Symptomen, Komplikationen oder Versagen der medikamentösen Therapie infrage. Hierbei ist das Ziel die Entfernung möglichst aller sichtbaren Herde und die Wiederherstellung physiologischer anatomischer Verhältnisse. Da Endometrioseherde auch auf extragenitalen Strukturen auftreten oder diese infiltrieren können, ist eine Kooperation von operativ tätigen GynäkologInnen und Allgemein‑/ViszeralchirurgInnen nicht selten sinnvoll, um optimale Ergebnisse zu erreichen. Dabei ist es einleuchtend, dass auch die allgemein‑/viszeralchirurgischen KollegInnen in der Lage sein müssen, endometriales Gewebe zu erkennen und erfolgreich zu resezieren [82]. Beteiligte Allgemein‑/ViszeralchirurgInnen sollten außerdem die möglichen Komplikationen kennen, die nach einer endometriosebedingten Op mit Darmresektion auftreten können, zu welchen neben vorübergehenden gastrointestinalen Beschwerden auch eine temporäre oder persistierende sexuelle Dysfunktion zählen kann [91].

In einigen Fällen kann sich die Endometriose präoperativ in Symptomatologie sowie klinischem Erscheinungsbild auch wie andere viszeralchirurgische Erkrankungen, z. B. ein Zäkumtumor oder auch das Rektumkarzinom, darstellen [24, 44, 78]. Werden die Patientinnen dann primär allgemein‑/viszeralchirurgisch vorstellig, gilt es insbesondere auch, eine Entscheidung darüber zu treffen, inwieweit Genitalanteile erhalten werden können. Auch hierfür ist fachliche Kompetenz vonseiten der Allgemein‑/ViszeralchirurgInnen gefragt [43] bzw. gynäkologischer Rat am Situs wünschenswert.

#### Entzündliche Erkrankungen

##### „Pelvic inflammatory disease“.

Primär gynäkologische entzündliche Erkrankungen können in einigen Fällen auch nichtgynäkologische Organe betreffen. So kann beispielsweise die „pelvic inflammatory disease“ (PID), welche häufig durch sexuell übertragbare Erreger hervorgerufen wird, zu Abszessen führen, die auch den Darm betreffen können. Eine Unterscheidung von zystischen Neoplasien kann bildgebend schwierig sein [86].

##### Chlamydieninfektion.

Ein weiteres Beispiel ist die durch *Chlamydia trachomatis* ausgelöste Primärinfektion. Während sich diese meist hauptsächlich im Genitalbereich abspielt, kann sie auch das Rektum betreffen und viszeralchirurgische Relevanz haben. Eine solche Infektion geht typischerweise mit inguinaler Adenopathie und Ulzerationen einher. Besonders bei später Behandlung können sich auch Fisteln, Abszesse oder Strikturen bilden [73, 86].

##### Gynäkologische Entzündungen mit extragenitalem Ursprung.

In einigen Fällen können Entzündungen gynäkologischer Organe einen allgemein‑/viszeralchirurgischen primären Fokus aufzeigen. So kommt es vor, dass sich bei Patientinnen, welche aufgrund einer Salpingitis operativ versorgt werden, intraoperativ ein entzündlicher Konglomerattumor darstellt, welcher den Darm einbezieht. In solchen Fällen besteht der eigentliche Fokus meist in einer gedeckt perforierten Sigmadivertikulitis oder einer akuten Appendizitis. Eine interdisziplinäre Kooperation unter Zuhilferufen allgemein‑/viszeralchirurgischer KollegInnen kann dann nötig sein, um über eine ggf. notwendige Darmresektion kompetent zu entscheiden und diese gegebenenfalls befundgerecht durchzuführen [43].

### Erkrankungsbilder mit viszeralchirurgischem Fokus

#### Maligne viszeralchirurgische Erkrankungen

##### Rektumkarzinom und die pelvine Exenteration.

Dem Rektumkarzinom kommt aufgrund seiner relativ hohen Inzidenz mit rund 50 % der kolorektalen Karzinome eine besondere Relevanz zu. Noch dazu ist die Ausbreitung des Rektumkarzinoms aufgrund der unterschiedlichen Metastasierungswege besonders komplex.

Für die interdisziplinäre Kooperation besonders relevant ist, dass es sich per continuitatem sowohl in urologische Organe (insbesondere in die Harnblase) als auch in gynäkologische Organe wie Vagina, Uterus und Adnexen ausbreiten kann [41].

Bei der pelvinen Exenteration handelt es sich um eine komplexe Op-Methode zur Behandlung rezidivierender oder lokal invasiver Malignome des Beckens mit dem Ziel der vollständigen Tumorentfernung [15, 39]. Hauptindikationen für diese Prozedur sind – nicht selten nach einschlägiger neoadjuvanter Vorbehandlung – das fortgeschrittene und andere Beckenorgane infiltrierende Rektumkarzinom bzw. dessen Rezidive. Doch auch bei weiteren malignen Tumoren wie dem des Endometriums, der Zervix oder der Ovarien kommt die pelvine Exenteration zum Einsatz [15, 39, 69, 81, 97]. Die totale pelvine Exenteration beinhaltet dabei die Resektion von Colon sigmoideum, Rektum, Anus, Harnblase sowie Urethra und gegebenenfalls knöcherner Strukturen. Zusätzlich erfolgt bei Männern die Resektion von Prostata und Bläschendrüse, während bei Frauen eine Adnexektomie, Hysterektomie und Vaginektomie zur Prozedur gehören [39].

Entsprechend der hohen Radikalität der pelvinen Exenteration handelt es sich um eine Intervention mit einem nicht unbeträchtlichen Komplikationspotenzial. So gibt beispielsweise eine systemische Literaturanalyse des „PelvEx Collaborative“ eine postoperative Morbidität von 53,6 % sowie eine Krankenhausletalität von 6,3 % an [68]. In einigen Patientengruppen ist zum Management der aufgetretenen Komplikationen bei bis zu einem Drittel der PatientInnen, welche mittels pelviner Exenteration therapiert wurden, eine weitere Intervention nötig [69]. Trotz dieser nicht unwesentlichen Komplikationsrate kann die pelvine Exenteration für bestimmte PatientInnengruppen nach verantwortungsvollem Abwägen von Nutzen und Risiko und interdisziplinärem Tumorboardentscheid jedoch eine valide Therapieoption sein, sodass diese auch elementarer Bestandteil der aktuellen Leitlinien ist [28, 50, 72, 81, 97].

Entsprechend der Komplexität des Eingriffs erfolgt die pelvine Exenteration häufig in interdisziplinärer Kooperation. Dabei können neben der Viszeralchirurgie und der Gynäkologie unter anderem auch die Urologie, Orthopädie und Neurochirurgie beteiligt sein [39]. Es erscheint einleuchtend, dass die Qualität der interdisziplinären Zusammenarbeit insbesondere bei dieser hochkomplexen und nicht gerade gering risikobehafteten Op einen hohen Stellenwert hat, bei der alle fachbezogenen Teammitglieder ihre spezifische Rolle in Aufgabenverteilung und Ablauf zu bestreiten haben.

##### Kolonkarzinom.

Auch beim Kolonkarzinom ist die synchrone sowie metachrone invasive Ausbreitung des malignen Tumors ausschlaggebend, welche häufig auch die gynäkologischen Organe, insbesondere die Ovarien, betrifft. Eine von einem Kolonkarzinom ausgehende Invasion der Ovarien kann dabei oftmals auch mit weiteren metastatischen Läsionen wie solchen des Peritoneums oder auch der Leber einhergehen, jedoch können die gynäkologischen Organe auch isoliert von anderen Metastasen betroffen sein. In Fällen mit ovarieller Beteiligung eines Kolonkarzinoms kommt es dabei nicht selten vor, dass die metastatische Läsion sogar deutlich größer ist als der Primarius [57]. Nicht zuletzt hierdurch sind von Kolonkarzinomen ausgehende ovarielle Metastasen in vielen Fällen nur schwer von primär ovariellen Tumoren zu unterscheiden, was eine besondere diagnostische Herausforderung darstellt [19, 48].

Spezifische Symptome können dabei häufig fehlen. Während die meisten Patientinnen als Initialsymptom über Schmerzen klagen, gaben in einer Studie in etwa nur 22 % der betroffenen Patientinnen uterine Blutungen als spezifisches Symptom an [57].

Zum Erreichen des kurativen Ziels der kompletten makroskopischen Tumorresektion ist bei Patientinnen mit auf die gynäkologischen Organe ausgebreiteten Kolonkarzinomen häufig eine extensive Resektion dieser sowie des Kolons und – je nach zusätzlicher Organbeteiligung – weiterer viszeralchirurgisch relevanter Organe in interdisziplinärer Zusammenarbeit notwendig [48, 57].

##### Krukenberg-Tumoren.

Neben den besprochenen Tumoren können auch weitere intraabdominelle Läsionen gynäkologische Organe miteinbeziehen. Eine besondere Form dieser Organbeteiligung ist die Ausbildung von Krukenberg-Tumoren. Hierbei handelt es sich um ovarielle Metastasen aus Siegelringzellkarzinomen. Während diese häufig von Magenkarzinomen ausgehen, können auch kolorektale Karzinome oder seltener auch maligne Tumoren unter anderem der Brust, der Appendix, des Dünndarms, der Gallen- und Harnblase den Ursprung für eine entsprechende Metastasierung darstellen [7, 62]. Krukenberg-Tumoren stellen in westlichen Ländern weniger als 4 % der diagnostizierten malignen Tumoren der Ovarien, jedoch zeigen sich dabei ausgeprägte regionale Unterschiede. In einigen asiatischen Ländern wie etwa in Korea, Japan und China machen Krukenberg-Tumoren beispielsweise etwa 20 % des Eierstockkrebses aus, was wahrscheinlich mit der hohen Prävalenz von Magenkarzinomen in diesen Regionen zusammenhängt [7]. Wichtiger Bestandteil der Therapie ist auch hier meist eine chirurgische Resektion der gesamten Tumormasse mit dem Ziel eines verlässlich erreichten R0-Status. Entsprechend des komplexen Krankheitsbildes mit Befall unterschiedlicher Organsysteme scheint es einleuchtend, dass auch hier eine interdisziplinäre Kooperation wünschenswert ist [7, 62].

#### Entzündliche Erkrankungen

Etliche entzündliche Erkrankungen der viszeralen Bauch- und Beckenorgane können – bereits aufgrund der Nähe zu gynäkologischen Strukturen – klinisch sowie auch in bildgebenden Untersuchungen wie eine Inflammation dieser imponieren. Weiterhin können sich entzündliche Prozesse von einem Organ ausgehend in andere ausbreiten, sodass sie letztlich mehrere Strukturen betreffen können. Im Folgenden soll der Fokus auf zwei in diesem Zusammenhang besonders relevante entzündliche Erkrankungsformen der Verdauungsorgane gelegt werden: die akute Appendizitis sowie chronisch-entzündliche Darmerkrankungen.

##### Akute Appendizitis.

Trotz rückläufiger Inzidenz handelt es sich bei der akuten Appendizitis nach wie vor um die häufigste Ursache für ein akutes Abdomen [1, 2, 46, 55, 67, 77]. Abgesehen von der bei ausgewählten Patientengruppen möglichen konservativ-medikamentösen Behandlung ist der Goldstandard der Therapie der akuten Appendizitis die operative Appendektomie, welche den häufigsten allgemein- und viszeralchirurgischen Notfalleingriff in Deutschland darstellt [74, 85]. In Deutschland zeigt sich seit einigen Jahren eine kontinuierliche Zunahme des Anteils an laparoskopisch durchgeführten Appendektomien, welche erstmals durch den Gynäkologen Semm durchgeführt sowie 1983 publiziert wurde und mittlerweile zum Standard der Behandlung geworden ist, selbst wenn sich das operativ-laparoskopische Herangehen auch dabei gewandelt hat [74, 77, 85].

Aus gynäkologisch-viszeralchirurgisch interdisziplinärer Sicht ist neben der Differenzialdiagnose gynäkologischer Erkrankungen mit nicht selten sehr ähnlicher Symptomatologie und klinischem Erscheinungsbild (Adnexitis, Salpingitis, Endometriose etc.) relevant, dass die akute Appendizitis auch andere Organe involvieren kann. Bilden sich beispielsweise von einer Appendizitis ausgehende Konglomerattumoren, besteht die Gefahr des Mitbefalls gynäkologischer Strukturen. In diesen Fällen kann eine interdisziplinäre Zusammenarbeit notwendig sein, um neben der Appendektomie auch die eventuelle Notwendigkeit einer Adnexektomie zu beurteilen oder, besonders bei jungen Frauen, die Fertilität durch zwingende Einbeziehung einer fachspezifischen Expertise (auch rund um die Uhr) möglichst zu erhalten [43].

##### Chronisch-entzündliche Darmerkrankungen.

Chronisch-entzündlichen Darmerkrankungen (CEDs) kommt nicht zuletzt aufgrund einer steigenden Inzidenz sowie der zunehmenden Komplexität der Therapie eine besondere Relevanz zu [60, 95, 98].

Dabei gibt es verschiedene Manifestationen der CEDs, welche gynäkologische Organe oder physiologische Funktionen betreffen.

Eine mögliche und belastende Erscheinung dieser Erkrankungen ist die der sexuellen Dysfunktion. Diese kann dabei – abhängig vom Schweregrad – von einer Einschränkung der Intimität und sexuellen Aktivität bis hin zur Erschwerung der Bildung und Aufrechterhaltung von Beziehungen führen. Zu den wichtigsten Risikofaktoren der sexuellen Dysfunktion im Rahmen von CEDs gehören dabei zum einen demografische Faktoren – wie in etwa das Alter – sowie vorbestehende psychische Erkrankungen, aber auch Medikamente, chirurgische Interventionen und die Krankheitsaktivität der zugrunde liegenden intestinalen Erkrankung [14, 30, 36, 71].

Insbesondere beim Morbus Crohn gibt es zudem eine Reihe begleitender Manifestationen an gynäkologischen Organen. Hierzu gehört die Ausbildung von Fisteln, welche sich – vom Darm ausgehend – auch in die weiblichen Geschlechtsorgane erstrecken können. Mit einem Anteil von 9 % an allen durch Morbus Crohn bedingten Fisteln ist die Häufigste dabei die rektovaginale Fistel. Trotz multimodaler Therapie erweist sich die Behandlung dieser Fisteln häufig als schwierig. Daher sollte hier eine interdisziplinäre Behandlung angestrebt werden [3, 30]. Neben der Vagina können sich im Rahmen des Morbus Crohn – vom Darm ausgehend – außerdem auch Fisteln in das Perineum, die Vulva, die Ovarien oder auch den Uterus ausbilden [30].

Zusätzlich zu Entzündungen, welche sich – *per continuitatem* vom Darm ausgehend – in andere Organe ausbreiten, existieren beim Morbus Crohn auch unabhängige extraintestinale Manifestationen, welche sich auch an gynäkologischen Organen finden lassen. So können sich granulomatöse Entzündungen gynäkologischer Organe ausbilden, welche sich beispielsweise im Sinne einer granulomatösen Salpingitis oder Oophoritis sowie als vaginale Granulome manifestieren können [30, 58, 88]. Als weitere seltene Manifestation lässt sich weiterhin die Ausbildung einer Entzündung der Vulva nennen. Die Schwere der Inflammation kann dabei von Erythemen und Ödemen der Labia majora über die Ausbildung von Granulomen bis hin zu schweren Ulzerationen und Abszessen reichen. Während diese Manifestation selten ist, kann sie die Lebensqualität der betroffenen Patientinnen einschränken – sie gilt weiterhin als unterdiagnostiziert [9, 20, 30, 32, 49].

Eine besondere Herausforderung in der Diagnostik der extraintestinalen Manifestationen von CEDs entsteht dadurch, dass diese häufig unabhängig von der intestinalen Krankheitsaktivität und sogar noch vor einem erkennbaren Befall des Darms auftreten können. Bei noch nicht bekannter CED gestaltet sich die Diagnose und die Ursachenklärung dementsprechend schwieriger [9, 49, 88].

Hinzu kommt außerdem eine Reihe weiterer Manifestationen, die eher indirekt mit der zugrunde liegenden CED zusammenhängen. Hierzu gehören unter anderem eine Dys- oder auch Amenorrhö sowie die Ausbildung von Bowen-Karzinomen an Vagina oder Vulva [30].

Weiterhin gibt es mögliche Interaktionen mit anderen Erkrankungen, welche im Rahmen von CEDs sowie deren Behandlung eine Relevanz haben. So hat sich beispielsweise gezeigt, dass einige der zur Therapie eingesetzten Medikamente das Risiko für maligne gynäkologische Tumoren der Zervix und der Vulva erhöhen können. Weiterhin besteht ein Zusammenhang zwischen CEDs sowie deren Therapie und einer erhöhten Rate an Infertilität sowie Endometriose, welche wiederum interdisziplinäre Relevanz haben kann (siehe oben; [89]).

Für in der Diagnostik und Therapie von CEDs involvierte ÄrztInnen ist es somit von Bedeutung, dass es sich bei diesen keineswegs um rein gastrointestinale Erkrankungen handelt. Krankheitsmanifestationen auch an gynäkologischen Organen sollten daher immer mitberücksichtigt werden.

#### Das akute Abdomen in der Schwangerschaft

Häufigste Ursachen für ein akutes Abdomen in der Schwangerschaft sind die akute Appendizitis, die akute Cholezystitis sowie der Ileus [6]. Die korrekte Diagnosestellung ist besonders in der Schwangerschaft eine Herausforderung. Gründe hierfür sind unter anderem der expandierende Uterus, welcher zu einer Lageverschiebung intraabdomineller Organe und damit zu Veränderungen der anatomischen Gegebenheiten und zu erschwerten Untersuchungsbedingungen führt, ebenso wie die ohnehin hohe Prävalenz der klassischen Symptomatik aus Übelkeit, Erbrechen und abdominellen Schmerzen auch bei gesunden schwangeren Frauen sowie eine im Allgemeinen zurückhaltende Positionierung bei der Indikationsstellung einer Op in der Schwangerschaft [6, 7, 61, 83]. Erschwerend kommt hinzu, dass während der Schwangerschaft nicht alle diagnostischen Verfahren in typischer Weise zum Einsatz kommen. So sollte mit Bildgebung im Sinne einer Computertomographie (CT) aufgrund der Strahlungsbelastung – soweit sinnvoll möglich – zurückhaltend umgegangen werden [47, 83], was den Anspruch an die dynamische Untersuchung der transabdominellen Sonographie und die Untersuchenden immens erhöht. Auch die Magnetresonanztomographie (MRT) kann als alternatives Verfahren besonders in der Schwangerschaft an Bedeutung gewinnen, bringt aber den Nachteil einer längeren Untersuchungsdauer mit sich und kann bei ausgeprägter Symptomatik daher ungeeignet [41, 77, 79] oder – vor allem im Bereitschaftsdienstsystem – nicht ausreichend verfügbar sein. Zusätzlich zur erschwerten Bildgebung können auch laborchemische Parameter im Zuge der Schwangerschaft oftmals verfälscht ausfallen – so sind eine Leukozytose oder eine Erhöhung der alkalischen Phosphatase beispielsweise nicht unüblich. Daher kann bereits diagnostisch ein operatives Vorgehen indiziert sein. Die diagnostische Laparoskopie kann in diesen Fällen eine Möglichkeit zur schnelleren, zuverlässigeren und sichereren Diagnosestellung darstellen [6, 87].

Auch therapeutisch kann – abhängig von der Ursache für die Symptomatik – eine großzügige Op-Indikationsstellung nötig sein [6, 7, 79]. Aufgrund während der Schwangerschaft deutlich reduzierter Kompensationsmechanismen kommt einer zügigen Entscheidung dabei eine besonders große Relevanz zu [79].

Die postoperative fetale sowie maternale Mortalität steigt im Allgemeinen mit der Schwangerschaftsdauer an und erreicht ihre Maxima häufig im 3. Trimenon. Neben einem engmaschigen Monitoring der fetalen Vitalparameter sollte dies auch bei der Frage nach der primären behandelnden Fachrichtung sowie bei postoperativen Kontrollen berücksichtigt werden. Besonders mit fortschreitender Schwangerschaft gewinnen eine gynäkologische Involvierung sowie Überwachung des Fetus zunehmend an Wichtigkeit.

##### Akute Appendizitis in der Schwangerschaft.

Wenngleich eine Schwangerschaft das Risiko einer akuten Appendizitis nicht *per se* zu erhöhen scheint, überschneiden sich die Prävalenzmaxima akuter Appendizitiden sowie von Schwangerschaften in Bezug auf die Altersverteilung. Hierdurch erklärt sich, dass die akute Appendizitis die häufigste Ursache für ein akutes Abdomen während der Schwangerschaft ist und dabei etwa 25 % der nichtgeburtshilflichen Op-Indikationen ausmacht [6]. Als spezifische Schwierigkeit in der Diagnostik einer akuten Appendizitis während der Schwangerschaft gilt, dass sich die Lokalisation der Appendix im Rahmen der fortschreitenden Schwangerschaft verlagern kann. Die Interposition des Uterus zwischen Appendix und Bauchwand sowie eine zunehmende Distension der abdominellen Muskulatur können das klinische Erscheinungsbild zudem beeinflussen. Hierdurch werden auch klinische Tests wie das Blumberg- oder das Psoas-Zeichen weniger aussagekräftig, stattdessen treten häufiger Flanken- oder Rückenschmerzen auf. Bei zudem häufig im Rahmen einer Appendizitis auftretenden Leukozyturie werden diese oft als Harnwegsinfekte oder Pyelonephritiden fehlgedeutet [6]. Auch die sonographisch gestützte Diagnostik wird im Laufe der Schwangerschaft zunehmend schwieriger. So beschreiben einige Studien, dass über 40 % der Frauen, die im 2. oder 3. Schwangerschaftstrimester appendektomiert wurden, tatsächlich histologisch unauffällige Appendizes aufwiesen [6, 40]. Verzögerungen der operativen Intervention erhöhen jedoch das Risiko von Perforation und Peritonitis und steigern hierdurch letztlich auch deutlich insbesondere die fetale Mortalität, sodass diese vermieden werden sollten. Minimal-invasive Eingriffe weisen – verglichen mit offenen Appendektomien – in Bezug auf die fetale Mortalität sowie die Frühgeburtlichkeitsrate ein geringeres Risikoprofil auf [6].

##### Akute Cholezystitis in der Schwangerschaft.

Die meist konkrementabhängige akute Cholezystitis stellt während der Schwangerschaft die zweithäufigste Ursache für ein akutes Abdomen dar. Eine progesteroninduzierte Relaxation der Gallenblasenmuskulatur führt hierbei zu einer Gallenstase und erhöht in Kombination mit hormongesteuerten Veränderungen der Gallezusammensetzung das Risiko der Bildung von Konkrementen [6, 10]. Die diagnostische Sensitivität des Murphy-Zeichens ist im Zuge der Schwangerschaft verringert, differenzialdiagnostisch müssen zusätzlich schwangerschaftsspezifische Pathologien wie das HELLP-Syndrom berücksichtigt werden. Therapeutisch kann durch eine primäre Operation häufig das Komplikationsrisiko gesenkt werden [6].

##### Ileus in der Schwangerschaft.

Dritthäufigste Ursache für ein akutes Abdomen während der Schwangerschaft ist der Ileus. Hierbei gilt als Besonderheit hervorzuheben, dass ein Volvulus bei schwangeren Ileuspatientinnen etwa 25 % der intestinalen Obstruktionen ausmacht. Bei nichtschwangeren Patientinnen ist ein Volvulus dagegen lediglich in 3–5 % der Fälle Auslöser des Ileus. Verglichen mit nichtschwangeren Patientinnen, ist die Mortalitätsrate im Rahmen eines Ileus bei schwangeren Patientinnen mit insgesamt 6 % deutlich erhöht und von der Schwangerschaftsdauer abhängig. Im 3. Trimenon kann sie bis zu 10–20 % erreichen, auch die fetale Mortalität ist im Rahmen der intestinalen Obstruktion deutlich erhöht. Therapeutisch unterscheidet sich das Vorgehen nicht wesentlich von nichtschwangeren Patientinnen, es sollte allerdings ein engmaschiges fetales Monitoring erfolgen [6, 70].

### Interdisziplinär relevante Komplikationen

Komplikationen können sowohl durch bestehende Erkrankungen direkt als auch iatrogen bedingt entstehen. Bei den mit einer Op in Zusammenhang stehenden iatrogenen Komplikationen kann weiterhin in intra- und postoperative Komplikationen unterschieden werden. Ein adäquates Komplikationsmanagement ist von enormer Bedeutung. Abhängig von der Entscheidung der Operierenden kann dabei auch bei primär gynäkologisch behandelten Patientinnen die Hilfe von Allgemein‑/ViszeralchirurgInnen notwendig werden [94]. Andererseits kann auch bei viszeralchirurgischen Eingriffen insbesondere an Kolon und Rektum bereits durch die anatomische Lagebeziehung eine Mitbehandlung der Urogenitalorgane notwendig werden. Bei entsprechenden präoperativen Befunden sollte daher bereits im Vorfeld der Op eine interdisziplinäre Abstimmung erfolgen, bei der das operative Vorgehen mit KollegInnen der Gynäkologie und Urologie im Rahmen eines interdisziplinären Forums besprochen wird [75], was für maligne Tumorerkrankungen mittels angezeigter fach-, organ- bzw. diagnosebezogener Tumorboards – vor allem für zertifizierte Tumorzentren – bereits hinreichend etabliert ist.

#### Intraoperative Verletzungen

Insbesondere im Rahmen gynäkoonkologischer Eingriffe sowie bei einer ausgedehnten Endometriose-Op kann es zu Darmverletzungen kommen [94]. Vorwiegend bei laparoskopischen Eingriffen können außerdem speziell Verletzungen des Dünndarms entstehen [51]. Abhängig von den situativen Gegebenheiten können Darmverletzungen genäht werden oder es kann eine Darmsegmentresektion notwendig werden [94]. Die Kooperation mit der Allgemein‑/Viszeralchirurgie im Rahmen einer gynäkologischen Op ist dann relevant, um eine zügige Entscheidung über das weitere Prozedere zu treffen und, wenn nötig, die entsprechende Versorgung zu übernehmen [25, 56, 94]. Dabei ist die frühzeitige Erkennung einer Verletzung ausschlaggebend für das Outcome der Patientinnen [51].

Andererseits können auch bei primär allgemein‑/viszeralchirurgischen Eingriffen die Genitalorgane verletzt werden. So kann es beispielsweise im Rahmen von Tumorresektionen zu unbeabsichtigten Verletzungen der Vagina kommen. Während kleinere Verletzungen meist ohne Probleme direkt von den (allgemein‑/)viszeralchirurgischen OperateurInnen genäht werden können, sollte vor allem bei größeren Verletzungen und sexuell aktiven Frauen die Einbeziehung der gynäkologischen KollegInnen dringend erwogen werden [75].

#### Unfallbedingte Verletzungen

**Figure 2:**
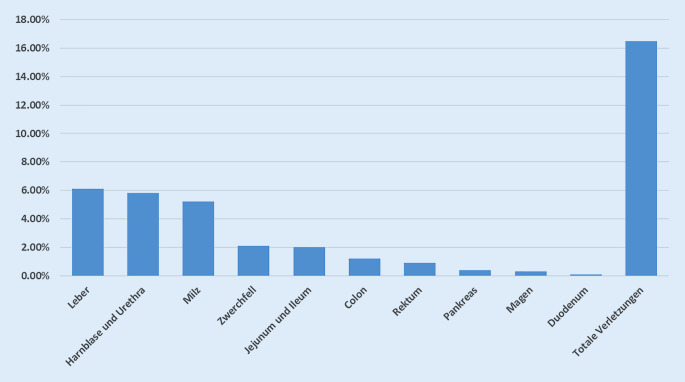

Neben iatrogenen Verletzungen der Bauch- und Beckenorgane im Zusammenhang mit diagnostischen oder therapeutischen Prozeduren ist auch eine traumatische Organverletzung möglich. Dabei können beispielsweise bei Beckenfrakturen oder Pfählungsverletzungen sowohl viszerale als auch gynäkologische Organe alteriert werden [21, 100]. Die am häufigsten betroffenen intraabdominellen Organe im Rahmen einer stumpfen Beckenfraktur sind die Leber (6,1 %), gefolgt von Harnblase und Urethra (5,8 %) sowie Milz (5,2 %; Abb. 2; [21]).

Verletzungen gynäkologischer Organe entstehen meist durch Einwirkung stumpfer Gewalt und betreffen zu etwa 75 % die Adnexe und zu etwa 25 % den Uterus [76]. Während ein Großteil dieser Verletzungen konservativ versorgt wird, kann in einigen Fällen auch eine operative Therapie notwendig werden. Hierbei erfolgt meist die anatomische und funktionelle Rekonstruktion der verletzten Organe, während nur in selteneren Fällen eine Resektion nötig ist [38, 76, 100].

Abhängig von den betroffenen Strukturen und Organen sowie vom Verletzungshergang erscheint es sinnvoll, dass eine operative Versorgung nach individueller Notwendigkeit und insbesondere im Falle einer kombinierten Verletzung intraabdomineller und genitaler Organe in interdisziplinärer Kooperation zwischen (Allgemein‑/)Viszeralchirurgie und Gynäkologie erfolgen sollte.

### Präoperative Diagnoseunsicherheit der prädominierenden Bildgebung

Die präoperative Einschätzung einer Erkrankung ist oftmals mit einer nicht unwesentlichen diagnostischen Unsicherheit behaftet, wodurch im Verlauf der Op unvorhergesehene Befunde ersichtlich werden können. So können beispielsweise Entzündungen verkannt werden oder Tumoren können sich weiter ausdehnen, als zuvor angenommen wurde, und eine multidisziplinäre Intervention notwendig machen [58]. Im Falle einer gleichzeitig zur Pathologie vorliegenden Schwangerschaft kann sich dabei die Diagnostik noch weiter anspruchsvoll erschweren [7]. In Folge kann sich bei einer ursprünglich als viszeralchirurgisch geplanten Op intraoperativ ein gynäkologischer Fokus zeigen, wie auch umgekehrt. In diesen Fällen ist häufig ein kurzfristiges Hinzuziehen einer interdisziplinären Beihilfe nötig.

#### Klinische Relevanz

Die nichtinvasive Bildgebung spielt in der Diagnosestellung diverser gynäkologischer sowie auch allgemein‑/viszeralchirurgischer Erkrankungen eine entscheidende Rolle. Aufgrund der hohen interdisziplinären Relevanz von Ovarialkarzinomen steht auch die Bildgebung dieser Entität im Mittelpunkt.

Beim Verdacht auf maligne Erkrankungen der Adnexe nimmt die transvaginale Sonographie den höchsten Stellenwert der bildgebenden Diagnostik ein. Die Abdominal- sowie Dopplersonographie sind ebenso für die Diagnostik und Einschätzung des Stadiums von Ovarialkarzinomen obligat [34].

Insbesondere im Hinblick auf das Staging und die Op-Planung erfolgt zudem häufig eine erweiterte Bildgebung. Diese kann eine Mammographie, CT oder MRT von Becken, Abdomen und Thorax sowie auch eine Positronenemissionstomographie (PET) umfassen [13, 34, 64].

#### Diagnostische Unsicherheit

##### Staging.

Die Sensitivität der transvaginalen Sonographie zur Erkennung eines Ovarialkarzinoms ist mit 95 % hoch. Dennoch können etwa 5 % der Ovarialkarzinome nicht durch diese erkannt werden [34]. Andere Studien konnten zeigen, dass die CT für das Staging des Ovarialkarzinoms mit Ausnahme des retroperitonealen Lymphknotenstatus zwar eine recht hohe Spezifität aufweist, die Sensitivität jedoch eher gering ist [64]. Im Vergleich konnte jedoch demonstriert werden, dass die MRT bei Patientinnen mit gynäkologischen Tumoren eine höhere Diagnosesicherheit bezüglich einer exakten Beschreibung der Tumorlokalisation und Ausdehnung bietet als die CT [16, 66]. In einer einzelnen Untersuchung waren hingegen selbst mit der MRT Abstriche hinsichtlich des präoperativen Befundes im Vergleich zum späterhin eruierten Befund zu machen [16].

Letztlich kann das Staging daher präoperativ durch die Bildgebung zwar unterstützt werden, ein nichtinvasives Verfahren, das beim Ovarialkarzinom das operative Staging ersetzen oder auch die Operabilität vollkommen verlässlich einschätzen kann, existiert jedoch nicht [34]. Auch bei anderen malignen Erkrankungen kann die Bildgebung allein in Bezug auf das Staging keine ausreichende Sicherheit bringen. So konnte beispielsweise gezeigt werden, dass synchrone Ovarialkarzinome bzw. ovarielle Metastasen im Rahmen eines primären Endometriumkarzinoms nicht immer bildgebend auffällig sind [27]. Auch beim Endometriumkarzinom ist die Referenzmethode für die lokale Ausbreitungsdiagnostik somit das operative Staging, welches bildgebend „nur“ unterstützt werden kann [28, 84].

##### Verwechslungsgefahren.

Zur Unsicherheit im Staging maligner Tumoren kommt eine Reihe von Erkrankungen hinzu, welche sich in der Bildgebung wie gynäkologische Tumoren darstellen können, jedoch tatsächlich anderen Ursprungs sind [26, 33].

Während die meisten Tumoren des weiblichen Beckens von den Genitalen ausgehen, lassen sich hier auch Tumoren anderer Abstammung finden. Beispiele sind unter anderem primär peritoneale, neurogene oder auch primär extraperitoneale Neoplasien. Auch gastrointestinale Prozesse (z. B. die bereits genannte akute Appendizitis, die akute [Sigma-]Divertikulitis und gastrointestinale Karzinome) können sich hier organüberschreitend oder -fern manifestieren. Die exakte Bestimmung des Ausgangspunktes von Läsionen kann sich in der Bildgebung schwierig gestalten. Aufgrund der komplexen Anatomie des weiblichen Beckens und der Ähnlichkeit der Merkmale wie klinische Zeichen und Symptomatologie verschiedener Pathologien kann sich die Diagnostik noch weiter erschweren, sodass solche Prozesse irrtümlicherweise mit primär gynäkologischen Läsionen verwechselt werden können [26, 33]. Ein Beispiel hierfür ist die ovarielle Metastasierung des Kolonkarzinoms, welche in einer Untersuchung in 45 % der Fälle als primär ovarielle Läsion verkannt wurde [19]. Hierdurch kann es intraoperativ zu von der präoperativ, insbesondere bildgebend erhobenen Diagnose abweichenden Gegebenheiten kommen, was dann eine primär unerwartete und prompte interdisziplinäre Kooperation in operativer Hinsicht nötig macht [43]. Die Fähigkeit zur Identifikation „regulärer“ weiblicher Genitalorgane spielt eine zentrale und bestimmende Rolle in der intraoperativen Differenzierung zwischen Ovarialtumoren und extraovariellen(/-genitalen) Tumorläsionen und ist somit auch für (Allgemein‑/)ViszeralchirurgInnen relevant [26].

### Viszeralchirurgische Eingriffe durch GynäkologInnen

Eine Vielzahl an Situationen im Rahmen gynäkologischer Op’s kann auch eine primär viszeralchirurgische Resektion erforderlich machen. Hier stellt sich die Frage, ob in solchen Fällen immer ein interdisziplinäres Vorgehen notwendig ist. Aktuelle Studien zeigen, dass gut ausgebildete GynäkoonkologInnen derartige Eingriffe prinzipiell auch ohne viszeralchirurgische Hilfe adäquat und mit guten Ergebnissen durchführen können [65]. Um dies zu gewährleisten, ist allerdings ein entsprechendes allgemein‑/viszeralchirurgisches Training im Rahmen der gynäkologischen Ausbildung notwendig. Befragungen von ÄrztInnen in Ausbildung zu GynäkoonkologInnen konnten dagegen zeigen, dass die Ausbildung in Bezug auf kolorektale Op’s – im Gegensatz zu den meisten primär gynäkologischen Interventionen – als unzureichend bewertet wurde [54].

### Organisatorische Aspekte der interdisziplinären Kooperation

Neben den rein medizinischen gibt es auch einige organisatorische Aspekte, welche für eine gute interdisziplinäre Zusammenarbeit von Bedeutung sind.

Um intraoperative Hektik oder kurzfristig planerischen Druck zu vermeiden, sollte eine bestmögliche präoperative Vorbereitung erfolgen. So sollte, wenn die Notwendigkeit einer interdisziplinären Zusammenarbeit absehbar ist, die jeweils andere Fachdisziplin bereits im Vorfeld hierüber informiert als auch möglichst für eine adäquate Abstimmung schon in die therapeutische Entscheidungsfindung und (operative) Therapieplanung einbezogen werden. Dabei ist konsequent die Möglichkeit zu nutzen, Befunde gemeinsam zu besprechen und Meinungen zur notwendigen Therapie und anstehenden Op auszutauschen. Wenn möglich, sollte dagegen ein kurzfristiges Hinzurufen der interdisziplinären KollegInnen erst während der Op weitestgehend vermieden werden, um die Vorbereitungsmöglichkeiten der KollegInnen auf den jeweiligen Fall zu verbessern und die Op- und Anästhesiezeit möglichst limitiert zu halten bzw. nicht unnötig zu verlängern.

Besonders für Notfälle, aber auch für Situationen, in denen die Notwendigkeit des Hinzuziehens der fachfremden Disziplin anhand der präoperativen Befunde nicht vorhersehbar war, ist es dagegen von besonderer Relevanz, konkrete Abläufe zu etablieren, welche dann ohne große Zeitverluste genutzt werden können, um intraoperative fachübergreifende Hilfe im Bedarfsfalle zeitnah hinzuzuziehen. Hierzu gehört, dass jedem Teammitglied bekannt sein sollte, welche Verantwortlichen in solch einem Fall zu kontaktieren und wie diese erreichbar sind, damit sie schnellstmöglich zur Unterstützung vor Ort sein können.

Voraussetzung für eine bestmögliche Zusammenarbeit ist dabei die Pflege der Kontakte zwischen den Fachrichtungen. KollegInnen sollten sich untereinander und die gegenseitigen Stärken gut kennen und aufeinander vertrauen, was insbesondere in Akutsituationen relevant ist, in denen zügig eine Entscheidung getroffen werden muss und ein schnelles Handeln ausschlaggebend ist.

### Stärken der Arbeit

Trotz der immer weiter fortschreitenden Spezialisierung und Subspezialisierung in der heutigen Medizin ist es wichtig, zusätzlich zum speziellen Fachgebiet auch ein breites Wissen zu verfolgen, das über die eigene Fachdisziplin hinausgeht. Bereits weil jegliche allgemein‑/viszeralchirurgische Erkrankung auch während der Schwangerschaft auftreten kann, ist es dabei besonders wichtig, dass ChirurgInnen sich stets im regen, zumindest periodischen Austausch mit ihren gynäkologischen KollegInnen befinden und die Einschränkungen und Schwierigkeiten kennen, die eine Behandlung während der Schwangerschaft mit sich bringt. Aber auch die Beteiligung gynäkologischer Organe bei allgemein‑/viszeralchirurgischen Erkrankungen sowie die präoperative Diagnoseunsicherheit führen dazu, dass auch Allgemein‑/ViszeralchirurgInnen jederzeit darauf vorbereitet sein müssen, mit gynäkologischen Fragestellungen konfrontiert zu werden, aber auch gendermedizinische Aspekte sukzessive je nach allgemein erzieltem Erkenntnisstand mit einzubeziehen.

Trotz dieser Umstände ist das Thema der interdisziplinären Zusammenarbeit im Allgemeinen nicht sonderlich quellenreich. Besonders im deutschsprachigen Raum sind kaum Arbeiten zu finden, die sich der Frage widmen, welches gynäkologische Wissen für Allgemein‑/ViszeralchirurgInnen relevant ist, welche Situationen erwartet werden können und auf welche Schwierigkeiten geachtet werden muss. In dieser Arbeit wird diese Frage mit eruiertem Neuheitswert des Anspruches aufgearbeitet.

### Limitationen

Während sich das vorliegende Manuskript auf diverse Aspekte der interdisziplinären Zusammenarbeit zwischen Allgemein‑/Viszeralchirurgie und Gynäkologie konzentriert, können andere interdisziplinäre Kooperationen hier nicht weiter ausgeführt werden. Zusätzlich zur Gynäkologie ist für die Allgemein‑/Viszeralchirurgie beispielsweise auch das interdisziplinäre Arbeiten mit der Urologie von besonderem Interesse (Manuskript in Vorbereitung). Obwohl einige der Aspekte wie die Notwendigkeit etablierter Abläufe zum Hinzuziehen einer intraoperativen Mithilfe oder auch die Relevanz der Interdisziplinarität im Umgang mit Komplikationen hierauf übertragbar sind, sollte auf spezifische Aspekte noch einmal gesondert eingegangen werden.

Nicht zuletzt blieben auf das weibliche Geschlecht bezogene spezifische gendermedizinische Aspekte nicht zuletzt aufgrund eines fest vorgegebenen Manuskriptumfangs komplett unberührt.

## Fazit

Das Gebiet der interdisziplinär ausgerichteten Interventionen in Kooperation von Allgemein‑/Viszeralchirurgie und Gynäkologie ist zumeist hochkomplex sowie in der aktuellen Literatur wenig erforscht und behandelt. Dabei reicht es von der präoperativen Zusammenarbeit zur Organisation gemeinsamer Prozeduren wie der pelvinen Exenteration bis hin zum fachüberschreitenden Management von spezifischen Fallkonstellationen oder Komplikationen wie der Verletzung des Intestinums während einer gynäkologisch-operativen Intervention. Hinzu kommt, dass jegliche üblicherweise allgemein‑/viszeralchirurgische Erkrankung auch bei schwangeren Frauen auftreten kann, was sowohl die Diagnostik als auch die Therapie erschweren kann und dann ggf. ein interdisziplinäres Vorgehen erforderlich macht. Bereits die anatomische Lagebeziehung von inneren Genitalorganen und Gastrointestinaltrakt macht darüber hinaus klar, dass von einer Organgruppe ausgehende Prozesse auch die jeweils andere involvieren können. Dies trifft dabei sowohl auf entzündliche als auch auf maligne Prozesse zu. In einigen Fällen kann sich aufgrund der präoperativen Restunsicherheit nach bildgebender Diagnostik auch eine komplette Abweichung vom geplanten Op-Prozedere ergeben, wobei auch ungeplant eine interdisziplinäre Zusammenarbeit zwischen Allgemein‑/Viszeralchirurgie und Gynäkologie notwendig werden kann.

Daher ergibt sich die hohe Relevanz, die Erkrankungsbilder der jeweils anderen Fachdisziplin zu kennen und (mit)beurteilen zu können. Allgemein‑/ViszeralchirurgInnen sollten auf den Fall vorbereitet sein, intraoperativ Pathologien der Genitalorgane vorzufinden, welche ggf. das Hinzuziehen eines Gynäkologen notwendig machen. Ebenso sollten sie darauf eingestellt sein, geplant oder auch ungeplant zu einer laufenden gynäkologischen Op hinzugerufen zu werden. Schließlich ergibt sich daraus auch die Bedeutung klar strukturierter Abläufe der interdisziplinären Kooperation, um im Falle der akuten Notwendigkeit einer interdisziplinären Unterstützung möglichst wenig Zeitverluste zu erleiden, kurzfristig qualifizierte KollegInnen hinziehen zu können und damit die Op- und Narkosezeit möglichst zu limitieren. Ansätze hierzu sind beispielsweise eine klare Aufgabenverteilung bezüglich der telefonischen Kommunikation im Op-Saal sowie die durchgehende Verfügbarkeit interdisziplinärer Konsiliare in der Nähe. Bei voraussehbarer Notwendigkeit einer interdisziplinären Zusammenarbeit sollte dagegen möglichst der gesamte Ablauf gemeinsam geplant werden. Dazu zählen eine gemeinsame Besprechung der diagnostischen Schritte, der primären Operateure und eine im Voraus für die Intervention geplante interdisziplinäre Präsenz sowie – abhängig von den zu erwartenden Komplikationen – die gemeinsame Festlegung der für die postoperative Überwachung am besten geeigneten Fachrichtung. Eine erhöhte interdisziplinäre Fachkompetenz der einzelnen Operateure kann dagegen auch das eigenständige Vorangehen erleichtern. Hierzu könnte ein erhöhter Fokus auf ebendiese operative Fertigkeiten im Rahmen der ärztlichen Weiterbildung, beispielsweise im Sinne viszeralchirurgisch-gynäkologischer Rotationen, sinnvoll sein.
